# Directly Observed Therapy to Measure Adherence to Tuberculosis Medication in Observational Research: Protocol for a Prospective Cohort Study

**DOI:** 10.2196/24510

**Published:** 2021-06-16

**Authors:** Elizabeth J Ragan, Christopher J Gill, Matthew Banos, Tara C Bouton, Jennifer Rooney, Charles R Horsburgh, Robin M Warren, Bronwyn Myers, Karen R Jacobson

**Affiliations:** 1 Section of Infectious Diseases Boston Medical Center Boston, MA United States; 2 Department of Global Health Boston University School of Public Health Boston, MA United States; 3 Departments of Epidemiology and Biostatistics Boston University School of Public Health Boston, MA United States; 4 Department of Science and Innovation–The National Research Foundation Centre of Excellence for Biomedical Tuberculosis Research and The South African Medical Research Council Centre for Tuberculosis Research Division of Molecular Biology and Human Genetics, Faculty of Medicine and Health Sciences Stellenbosch University Cape Town South Africa; 5 Alcohol, Tobacco and Other Drug Research Unit South African Medical Research Council Cape Town South Africa; 6 Division of Addiction Psychiatry Department of Psychiatry and Mental Health University of Cape Town Cape Town South Africa

**Keywords:** tuberculosis, directly observed therapy, treatment adherence and compliance, medication adherence, mobile applications

## Abstract

**Background:**

A major challenge for prospective, clinical tuberculosis (TB) research is accurately defining a metric for measuring medication adherence.

**Objective:**

We aimed to design a method to capture directly observed therapy (DOT) via mobile health carried out by community workers. The program was created specifically to measure TB medication adherence for a prospective TB cohort in Western Cape Province, South Africa.

**Methods:**

Community workers collect daily adherence data on mobile smartphones. Participant-level adherence, program-level adherence, and program function are systematically monitored to assess DOT program implementation. A data dashboard allows for regular visualization of indicators. Numerous design elements aim to prevent or limit data falsification and ensure study data integrity.

**Results:**

The cohort study is ongoing and data collection is in progress. Enrollment began on May 16, 2017, and as of January 12, 2021, a total of 236 participants were enrolled. Adherence data will be used to analyze the study’s primary aims and to investigate adherence as a primary outcome.

**Conclusions:**

The DOT program includes a mobile health application for data collection as well as a monitoring framework and dashboard. This approach has potential to be adapted for other settings to improve the capture of medication adherence in clinical TB research.

**Trial Registration:**

Clinicaltrials.gov NCT02840877; https://clinicaltrials.gov/ct2/show/NCT02840877

## Introduction

A major challenge for prospective clinical tuberculosis (TB) research is accurately defining a metric for measuring medication adherence [1,2]. Accurate measurement of adherence is imperative to correctly capture a medication’s impact and guide best dosing, including the thresholds for missed doses and exposure impact on toxicity [3]. Inaccurate adherence characterization can lead to the misinterpretation of study findings, including those of drug efficacy [4]. From self-report to direct observation, researchers grapple with weighing cost and privacy with efficiency and accuracy when considering methods for adherence capture.

Medication adherence and preventing loss to follow-up in TB treatment are critical for individual favorable treatment outcomes, preventing recurrence, and stopping forward transmission [5]. Directly observed therapy (DOT) has been central to TB treatment programs since the World Health Organization (WHO) adopted the directly observed therapy, short-course (DOTS) strategy in 1994 [6]. Since the initiation of the 2015 End TB Strategy, the WHO has highlighted the potentially important role that digital solutions can play in supporting medication adherence [7]. The adherence technologies currently available on the market have been summarized elsewhere [8]. Although these approaches, such as electronic drug monitoring, have been widely trialed for HIV and have been increasingly implemented programmatically for TB, application in prospective TB research has been limited [9].

Electronic approaches to adherence monitoring are not without their challenges in the context of TB treatment. Recently, electronic drug monitoring has been studied for TB treatment adherence. 99DOTS, an adherence program launched in India in 2016, has shown mixed results [10-13]. Medication Event Monitoring Systems has also been associated with mixed reliability and efficacy [14-16], and video-observed therapy and wirelessly observed therapy [17,18] may face implementation challenges in low-resource settings. Another ongoing project in India, Operation ASHA, uses biometric fingerprint scanning at centralized TB treatment centers to confirm adherence, but preliminary qualitative results indicate positive adherence behavior change in only about half of patients [19]. Given the limitations of certain electronic adherence monitoring approaches (eg, Medication Event Monitoring Systems) and the cost and challenges of implementing strategies such as video-observed therapy in resource-constrained settings, we sought to combine the approaches of in-person adherence monitoring and electronic monitoring to facilitate accurate measurement of medication adherence in the research setting.

The Tuberculosis Treatment Outcomes and Alcohol Use Study (TRUST; R01AI119037) [20] is a prospective cohort study which aims to investigate the association between problem alcohol use and TB treatment response, controlling for medication adherence. A critical component of the study design is high-quality measurement of adherence in order to enable accurate measurement of the relationship between alcohol exposure and treatment response. Here we present the design and methodology of a mobile health (mHealth)–based DOT program developed for the purpose of measuring anti-TB medication adherence for TRUST participants.

## Methods

### Study Setting

The TRUST study recruits newly diagnosed TB patients receiving treatment at a primary health clinic in Worcester, the fourth largest city in South Africa’s Western Cape Province. The subdistrict from which the study recruits from has one of the highest TB burdens in the region (2016 incidence rate of 933 per 100,000 population; D Theron, personal correspondence to K Jacobson, 2017) and concurrent high rates of alcohol use [21-23]. The standard of care for anti-TB medication adherence monitoring at the study clinic is a variant of DOT in which patients choose a family member or friend who commits to observing their daily dose and signing a small card as confirmation. Patients bring this card to their monthly follow-up visit to be reviewed by a clinic nurse and documented within the medical record. This approach is highly vulnerable to falsification and misreporting. To improve this, TRUST developed and implemented the following program.

### TRUST DOT Program Overview

The TRUST DOT program operates fully independent of the clinic’s TB program and current DOT activities. A team of professional community workers (DOT workers) is overseen by TRUST’s dedicated DOT coordinator and community liaison. Our program’s innovation is that DOT workers collect adherence data on smartphones, submitting forms for each of their assigned participants back to an encrypted central server each weekday (see details in Mobile Data Collection Application). DOT workers scan a unique quick response (QR) code affixed to the participant’s medication box at each encounter to confirm being in the presence of the participant. The date, time, and GPS coordinates of the encounter are captured each time a form is submitted. A separate form is submitted at the beginning of each week to capture self-reported adherence for the preceding Saturday and Sunday.

DOT workers live within the study clinic’s catchment area and are assigned participants who live in the same neighborhoods as themselves for ease of conducting daily visits. DOT workers are organized into “neighborhood groups,” allowing for case sharing within each group through the mobile app. Participants are initiated on DOT within 1 week of starting TB treatment. At the initiation visit, the DOT coordinator and assigned DOT worker visit the home of the participant, register them to the system, and review participant expectations. The DOT worker then visits the participant every weekday until either treatment completion or end of study participation, whichever comes first.

### Program Definitions

DOT workers observe dosing Monday through Friday (defined as “DOT days”) with participants self-reporting their weekend adherence data. Routine adherence and program monitoring focuses on DOT days, with periodic data audits addressing weekends. Participants currently enrolled in the study and on treatment are considered active cases. Optimal adherence to anti-TB medication is often cited as 90% [24]. This measure has been used in multiple studies to classify good or optimal adherence [25-28]. Informed by this threshold, we defined 3 adherence tiers to capture the degree to which the participant has diverged from optimal adherence (Table 1). DOT workers record both observed and self-reported adherence on DOT days. Observed adherence is defined as a DOT worker or other study team member (eg, study nurse) personally observing the participant take their medication. Self-reported adherence is defined as the participant reporting that they took their medication. A participant is classified as nonadherent on a day when they cannot be located or reached or if they report not taking their medication.

Additionally, we defined 3 participation tiers to describe the degree to which a participant is engaging with the DOT program. This is assessed by looking at the proportion of DOT days in which adherence was self-reported versus observed, with a higher proportion of days in which adherence was self-reported indicating diminishing participation in the study (Table 1).

**Table 1:** Directly observed therapy adherence tiers for program participation monitoring.

Tiers	Green	Yellow	Red
Adherence tiers	Adherent on ≥90% of DOT^a^ days	Adherent on 75%-89% of DOT days	Adherent on <75% of DOT days
Participation tiers	Reported adherence on <20% of DOT days	Reported adherence on 20%-39% of DOT days	Reported adherence on ≥40% of DOT days

^a^DOT: directly observed therapy.

### Mobile Data Collection App

Daily DOT data are collected and submitted on basic smartphones carried by each DOT worker. The TRUST DOT data collection app was built using CommCare, an open source mHealth platform for electronic data collection and case management [29] and is outlined in Multimedia Appendix 1. DOT workers log in and submit data through unique user accounts. Every DOT worker in a given neighborhood group can access all active cases registered within that group through their active case list. The DOT coordinator and each study staff member can access all active cases across all neighborhood groups from their own user account.

**Figure 1:**
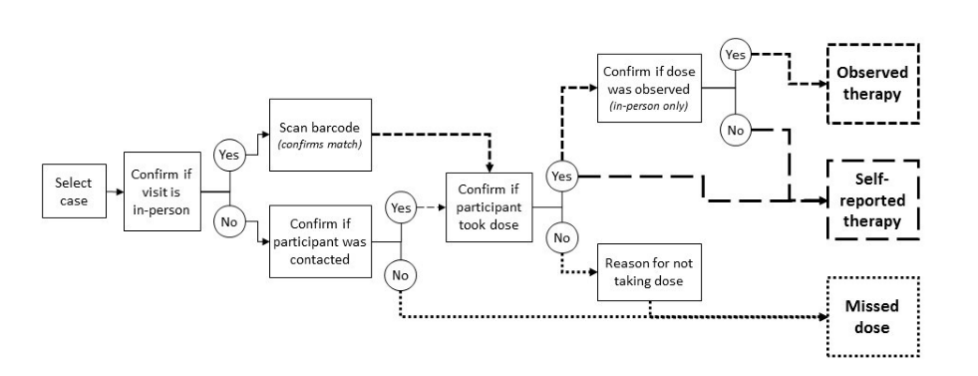
Flow diagram of data collection app for directly observed therapy days (Monday through Friday). Small dashed lines denote pathways to observed therapy. Large dashed lines denote pathways to self-reported therapy. Dotted lines denote pathways to a missed dose classification.

The app structure is described in Figure 1. When conducting a daily visit, DOT workers confirm that they are with the participant by using the app to scan a unique QR code that study staff affix to the participant’s medication box. A new QR code sticker is affixed to each box of medication dispensed by the TB program at monthly clinic visits. The app cross-checks the scanned QR code against the QR code registered for the given participant in the system and only proceeds if a match is confirmed. The DOT worker then indicates whether the participant took their medication and whether the dose-taking was observed (ie, the DOT worker may meet with a participant in-person, but the participant could have taken their dose before the DOT worker arrived). The DOT worker may proceed through the app without scanning a QR code, but observed adherence is not considered validated unless a QR code has been scanned. Alternatively, the DOT worker may select that they were not with the participant and then indicate whether they received an indirect report that the participant took their medication.

### Adherence and Program Monitoring Indicators

For purposes of systematically monitoring the DOT program, we defined indicators to assess both individual participant-level adherence as well as program-level adherence and program function (Textbox 1).

**Indicators for the directly observed therapy program and participant performance and engagement.**

**Participant-level adherence monitoring indicators**

1. What adherence tier does the participant fall into?
2. What participation tier does the participant fall into?
3. Has the participant been nonadherent on 2 or more days of their last 5 directly observed therapy (DOT) days?
4. Has the participant had no observed therapy for 2 or more of their last 5 DOT days?

**Program-level monitoring indicators**

1. What proportion of participants fall into each adherence tier?
2. What proportion of participants fall into each participation tier?
3. What proportion of DOT workers observed therapy for ≥80%, 60%-79%, and <60% of their participants in the last 5 DOT days?
4. What proportion of each DOT worker’s participants have observed therapy for ≥90%, 75%-89%, and <75% of the DOT days?

#### Individual Participant-Level Indicators

Participant-level adherence indicators assess both recent and cumulative trends in adherence and DOT program participation for each study participant. Suboptimal adherence (whether recent or cumulative) or an increase in self-reported adherence alert study staff to a participant’s decreased engagement and potential risk of missing further doses and subsequent loss to follow-up. When assessing recent adherence, we look at DOT days as continuous from Monday to Friday.

#### Program-Level Indicators

We also developed indicators to examine program-level trends. At the program level, we assess the proportion of participants falling into each adherence and participation tier. At the DOT worker level, we assess adherence trends of all participants assigned to a given DOT worker and compare trends across DOT workers. Variability across DOT workers alerts study staff to potential issues in worker performance. Trends across all study participants alert staff to program functionality or adherence trends across the study population.

### Data Monitoring and Visualization

We created a dashboard in Microsoft Excel (Microsoft Corporation) to display data against participant- and program-level indicators and which is provided in Multimedia Appendix 2. The study’s data dashboard is linked to the CommCare server and, when opened while connected to internet, actively pulls and updates all data submitted up until the day prior. Reflective of the aforementioned monitoring indicators, the dashboard is organized into 2 tabs: (1) participant monitoring and (2) DOT worker monitoring. Both tabs are filterable by date.

#### Participant Monitoring Tab

In the participant monitoring tab, data for active cases are aggregated and displayed at the participant-level. Participants are selected based on their unique study ID and registered case name from a scrolling list. For each participant, adherence information from the last 7 consecutive DOT days is displayed in a table along with cumulative adherence and cumulative percent of adherence observed. Multiple sorting buttons allow the user to subset the list of those participants with a cumulative adherence ≤75%, those with a cumulative self-reported adherence on ≥40% of DOT days, those who missed 2 or more of their last 5 DOT days, and those who had no observed therapy for 2 or more of their last 5 DOT days.

#### DOT Worker Monitoring Tab

In the DOT worker monitoring tab, data are aggregated by worker. A table displays the number and proportion of participants in each adherence tier by DOT worker. When a cell displaying a given adherence tier for a specific DOT worker is selected, all participants that fall into that cell are displayed within a scrolling menu. When a participant is selected from this menu, their adherence for the last 7 consecutive DOT days is displayed in a table.

### Program Implementation

Before implementation, DOT workers undergo hands-on training on using the mobile app, engaging with participants, and conducting good clinical practice. DOT workers are trained to remain impartial to whether or not a participant takes their medication and are instructed to focus on encouraging the participant to meet and communicate with them in person, regardless of adherence. Follow-up on suboptimal adherence is the responsibility of the study’s DOT coordinator and the clinic. As per agreement with the clinic, information on participant adherence is reported back to the clinic nurses who decide the appropriate course of action. The study’s goal is to collect adherence accurately, not to change or improve medication adherence patterns.

At the initial study visit, the study nurse schedules a DOT initiation visit for each participant within 1 week of their enrollment. The study nurse places a unique QR code on the participant’s pillbox that has the participant’s unique study ID embedded within it. This is scanned by the DOT worker at each DOT visit to confirm they are with the participant. Participant details and the scheduled DOT initiation date are communicated to the DOT coordinator, who assigns a DOT worker to the participant based on where the participant lives and the current caseloads of the DOT workers.

At the DOT initiation visit, the DOT coordinator meets the assigned DOT worker at a location agreed upon with the participant, typically the participant’s home. At this time, the DOT coordinator goes over the expectations of the participant and the DOT worker together. A routine time and place for DOT is scheduled, and the participant is registered as an active case on the DOT worker’s phone.

As mentioned previously, DOT workers are each assigned to 1 of 3 neighborhood groups. DOT workers communicate regularly with one another and the DOT coordinator so that should one worker be unavailable, cases can easily be reassigned to ensure visits are not missed. For instance, if a participant needs to be admitted to the hospital, the DOT coordinator can submit forms for all active cases across all neighborhood groups.

DOT workers are required to submit 1 form per participant per weekday. If they are unable to meet or contact a particular participant on a given day, they submit a form indicating so and the participant is categorized as nonadherent on that day. No weekday adherence data are submitted retrospectively.

When a participant completes treatment or ends study participation, whichever comes first, DOT visits end and the DOT coordinator closes the participant’s case through the app.

### Preventing Data Fabrication

Data fabrication is a risk faced by all field research; we implement numerous measures to prevent this. First, reporting observed adherence requires the DOT worker to scan a QR code on the participant’s pillbox, which stays in the possession of the participant. Second, the phone’s GPS coordinates are automatically recorded by the app each time a form is submitted. Although the exact location of DOT visits may vary, trends and outliers may indicate falsification of data. If there is concern that a DOT worker may be fabricating data, these GPS coordinates can be cross-referenced against where the DOT worker is expected to be at the time of a DOT visit. Third, routine and regular monitoring of submitted data through the dashboard and through larger data audits allows for identification of trends of nonadherence and trends of perfection, which when clustered with a specific DOT worker and inconsistent with the programwide trends, may indicate falsified data.

DOT workers attend a weekly meeting with the study DOT coordinator, where they discuss challenges and receive feedback based on data submitted. Ongoing refresher trainings are conducted at these regular meetings. Finally, the DOT coordinator conducts regular scheduled and unscheduled observations of DOT workers during their daily visits.

### Ethical Considerations

This project is approved by the Boston University/Boston Medical Center Institutional Review Board (protocol no. H-34970; approved March 17, 2016) and the ethics committees of the South African Medical Research Council (protocol no. EC011-5/2016, approved July 5, 2016), Stellenbosch University (protocol no. SU-BEE17-0001; approved May 5, 2017), and University of Cape Town (protocol no, 497/2016; approved July 13, 2016). The study is also approved by the South African Western Cape Department of Health.

## Results

### Recruitment and Enrollment

The study began recruitment on May 16, 2017, and recruitment is ongoing. As of January 12, 2021, a total of 236 TB patients have been enrolled into the TRUST cohort. Of these participants enrolled, 206 remained enrolled in the study throughout the duration of TB treatment. A total of 21 were not followed up through treatment completion due to withdrawal from the study (n=7, 33.3%), moving out of the study catchment area (n=5, 23.8%), treatment failure (n=1, 4.8%), death (n=2, 9.5%), or unspecified reasons (n=5, 28.6%). A total of 20,789 forms recording medication adherence have been submitted via the mobile app.

### Planned Analyses

Following the closing of enrollment and conclusion of participant follow-up, investigators will perform primary analyses to model predictors of time to mycobacterial culture positivity—a marker of treatment response—and treatment outcomes, while controlling for adherence. Additional secondary analyses with adherence as the primary outcome will be performed to assess the performance of this DOT program and patterns of adherence and to better understand TB treatment adherence overall. Details on the assessment of the study endpoints are described further in the published study protocol [20].

## Discussion

We describe an mHealth-based DOT program to collect anti-TB medication adherence information in the context of a prospective cohort study. Accurate measurement of medication adherence as part of prospective clinical research is paramount to successfully conducting quality research on mechanism and effect. Due to inaccurate and nonstandardized measurements in clinical research, we do not know the accurate impact of TB medication adherence on treatment response. With poor capture, adherence remains an unmeasured potential confounder of other factors associated with poor outcomes. As mHealth interventions gain popularity for improving TB outcomes, an improved knowledge of TB medication adherence and a gold standard method to capture adherence data are needed to assess these interventions [7,30].

We recognize a limitation of our approach is the reliance on self-reported adherence for weekends. Binge drinking, particularly on weekends, is the main pattern of alcohol consumption within our study population [23]. Adherence on weekends during heavy drinking episodes may be lower and therefore vulnerable to the weaknesses of self-reporting. Another limitation is considering a missed day as a missed dose. Because participants remain in possession of their medication, they may take their medication on days when a DOT worker is unable to connect with them, including on holidays when our DOT workers do not work, or during periods of community violence or disruption, leading to underestimation. Additionally, while the scanning of a QR code confirms colocation of the study participant and DOT worker, it does not ultimately confirm that the medication was swallowed. This is a limitation that strategies such as video-observed therapy attempt to overcome. It is important to note that the DOT workers employed by this study are in no way incentivized by their assigned participants’ adherence and are instead encouraged to record accurately whether a dose was taken or not: the intervention for nonadherent patients is the responsibility of the study nurses, not the DOT workers. Scalability of this outlined program would depend on the resources available to a given project and the geographical catchment area of a given study.

We present a model for how to leverage mHealth technology to maximize efficiency and validity of community-based DOT in prospective clinical TB research. Additionally, real-time adherence and engagement monitoring may empower study teams to act swiftly to prevent disengagement or loss from care and potential treatment failure due to medication nonadherence. The details in this approach have the potential to be adapted for other prospective TB research studies in order to improve capture of medication adherence outside of a hospitalized setting.

## Appendix

Multimedia Appendix 1 Outline of the CommCare directly observed therapy mobile phone app.

Multimedia Appendix 2 Microsoft Excel directly observed therapy data dashboard.

## Abbreviations

DOT: directly observed therapy
DOTS: directly observed therapy, short-course
mHealth: mobile health
QR: quick response
TB: tuberculosis
TRUST: Tuberculosis Treatment Outcomes and Alcohol Use Study
WHO: World Health Organization
