# Supplementary material for: Directly Observed Therapy to Measure Adherence to Tuberculosis Medication in Observational Research: Protocol for a Prospective Cohort Study
Source: JMIR Res Protoc. 2021 Jun 16;10(6):e24510. doi: 10.2196/24510 (PMC8277341; doi:10.2196/24510)
Supplement: Multimedia Appendix 1 [file resprot_v10i6e24510_app1.docx]

**Supplement 1:** Outline of the CommCare DOT mobile phone application

Below is the outline of the DOT mobile phone application for the TRUST study, built on Dimagi Inc.’s CommCare platform (<https://www.dimagi.com/commcare/>). Black text indicates what the app user sees. Blue text indicates the response set/type. Red text provides details about the given question, including if it is a required field, its purpose/function, etc. Text in [brackets] indicates text that is piped from previous questions (e.g., participant name). Each numbered step indicates a new field in the given form of the CommCare application.

The application is set up to allow *case sharing,* a CommCare feature that allows the defining of user groups (i.e., our DOT neighborhoods) and the sharing of cases (i.e., DOT participants) between users within that group.

**Module 1: DOT Registration**

**Registration Form**

*This form is used to create a new case, i.e., register a new DOT participant to the DOT program. Note that with case sharing enabled, the only users who are able to register new cases (i.e., complete this form) are those who are members of all user groups (e.g., DOT coordinator, study nurse, etc.).*

1. This form registers new DOT participants.
2. What is the participant's first name?
   1. (free text)
   2. The response to this question is defined as #form/Name, and is piped throughout the rest of the application as indicated by [participant name]
3. Please scan [participant name]'s ID barcode, located on the back of their pill box.
   1. (barcode scanner)
   2. This is a required field
   3. This field is defined as #form/Patient_ID, and is used to validate barcode scanning in the DOT Follow Up module.
4. [participant name]'s participant ID is: [PID]
   1. This field pulls the PID from the previous field (#form/Patient_ID) to allow the user to visually confirm that the PID is correct
5. Did you set up a time and location to regularly meet with [participant name]?
   1. Yes/No
   2. This is a required field

**Module 2: DOT Follow Up**

**Weekday Form**

*This form is the primary DOT visit form, and is used to capture adherence information for a regular weekday DOT visit.*

1. This is a follow-up form for daily DOTS.
2. Date of visit
   1. (date)
   2. This is a required field
3. Did you meet with [participant name] in-person today?
   1. Yes/No
   2. This is a required field
4. Scan [participant name]'s pill bottle barcode.
   1. (barcode scanner)
   2. This field matches the barcode scanned against the initial barcode scanned during registration (i.e., #form/Patient_ID)
   3. Only appears if response to Question #3 is “Yes”
5. Did you get in touch with [participant name] today?
   1. Yes/No
   2. This is a required field
   3. Only appears if response to Question #3 is “No”
6. Did [participant name] take their medication today?
   1. Yes/No
   2. This is a required field
   3. Only appears if response to Question #3 is “Yes” or Question #5 is “Yes”
7. Did you observe [participant name] taking their medication today?
   1. Yes/No
   2. Only appears if response to Question #3 is “Yes” and Question #6 is “Yes”
8. What time did [participant name] take their medication today?
   1. (time)
   2. Only appears if response to Question #6 is “Yes”
9. Why did [participant name] not take their medication today?
   1. Too ill
   2. Refused
   3. Other
   4. Don't know
   5. Only appears if response to Question #6 is “No”
10. This is the end of the form.

**Weekend Form**

*This form is used to record self-reported weekend adherence, and is typically completed on Monday of each week during the regular weekday DOT visit.*

1. This form allows you to record if [participant name] took their medication over the weekend.
2. Are you meeting with [participant name] in person today?
   1. Yes/No
   2. This is a required field
3. Scan [participant name]'s pill bottle barcode.
   1. This field matches the barcode scanned against the initial barcode scanned during registration (i.e., #form/Patient_ID)
   2. Only appears if response to Question #2 is “Yes”
4. What was last Saturday's date?
   1. (date)
   2. This is a required field
5. Did [participant name] take their medication last Saturday?
   1. Yes/No/Don’t know
   2. This is a required field
6. What was last Sunday's date?
   1. (date)
   2. This is a required field
7. Did [participant name] take their medication last Sunday?
   1. Yes/No/Don’t know
   2. This is a required field
8. This is the end of the form.

**Close-Out Form**

*This form is used to permanently close out a case, and is typically completed either when a DOT participant completes treatment or when they are lost to follow-up.*

1. This form permanently closes a case.
2. Confirm case close
   1. Yes/No
   2. This is a required field
3. Close Reason
   1. End of follow-up
   2. Lost to follow-up
   3. Other
   4. This is a required field
